# Selection of DNA aptamer and its application as an electrical biosensor for Zika virus detection in human serum

**DOI:** 10.1186/s40580-022-00332-8

**Published:** 2022-09-10

**Authors:** Goeun Park, Myoungro Lee, Jiatong Kang, Chulwhan Park, Junhong Min, Taek Lee

**Affiliations:** 1 Department of Chemical Engineering, Kwangwoon University, 20 Kwangwoon-Ro, Nowon-Gu, Seoul, 01897 Republic of Korea; 2 School of Integrative Engineering, Chung-Ang University, Heukseok-Dong, Dongjak-Gu, Seoul, 06974 Republic of Korea

**Keywords:** Zika virus, Electrical biosensor, MXene, Aptamer, SELEX

## Abstract

Zika virus is a highly infectious virus that is part of the flavivirus group. Precise diagnosis of the Zika virus is significant issue for controlling a global pandemic after the COVID-19 era. For the first time, we describe a zika virus aptamer-based electrical biosensor for detecting Zika virus in human serum. The electrical biosensor composed of a Zika virus aptamer/MXene nanoparticle heterolayer on Au micro-gap electrode (AuMGE)/print circuit board (PCB) system. The Zika virus aptamer was designed to bind the envelope protein of the Zika virus by systematic evolution of ligands by exponential enrichment (SELEX) technique. The binding affinity of the aptamer was determined by fluorescence. For improving the sensor signal sensitivity, Ti_3_C_2_T_x_ MXene was introduced to surface of Au micro-gap electrode (AuMGE). The immobilization process was confirmed by atomic force microscopy (AFM). The prepared aptamer/MXene immobilized on AuMGE can detect the Zika virus through capacitance change according to the target concentration. The capacitance signal from the biosensor increased linearly according to increment of envelope proteins in the human serum. The limit of detection was determined to 38.14 pM, and target proteins could be detected from 100 pM to 10 μM. Thus, the developed electrical aptabiosensor can be a useful tool for Zika virus detection.

**Supplementary Information:**

The online version contains supplementary material available at 10.1186/s40580-022-00332-8.

## Introduction

Zika is a mosquito-borne virus of the genus *Flavivirus* which also include viruses such as yellow fever, dengue fever, and West Nile. Zika virus was discovered in 1947 and was first recognized as a human disease when a viral infection was confirmed in humans in Nigeria in 1953 [1]. It has been known to occur in Asia and Africa since the 1950s but has since spread to the Americas, where the Zika virus was prevalent from 2015 to 2016 [2]. In the future, there is a possibility that the affected area will expand due to changes in the vector ecosystem caused by global warming, crowd immunity, and urbanization [3]. In addition, as globalization due to post-corona starts and exchanges between countries occur, the number of mosquito-borne infectious diseases imported from abroad will increase. Most Zika virus infections are asymptomatic (50–80%), and symptomatic infections are mild and present with mild fever and muscle pain [4]. Additionally, the disease can be transmitted through non-veterinary mechanisms such as blood transfusions and sexual contact as well as from mother to fetus, causing microcephaly [5]. Moreover, co-infection of Zika and dengue viruses is dangerous and causes acute tissue damage and transient anemia [6]. Therefore, if a visitor to an area where Zika virus is prevalent shows suspicious symptoms, a means to determine whether the individual is infected is required.

Methods of diagnosing Zika virus infection include detection of viral nucleic acid by reverse-transcription polymerase chain reaction (RT-PCR) [7], detection of virus by electrochemical measurement method [1], and detection of IgM antibodies by IgM-capturing enzyme-linked immunosorbent assay (MAC-ELISA) [1]. Since antibodies against the Zika virus can cross-react with the dengue virus, MAC-ELISA can show positive results for dengue [1]. The plaque reduction neutralization test (PRNT) can distinguish antibodies from these closely related viruses and can be used to confirm the results of MAC-ELISA [9]. However, PRNTs are expensive, slow, and require reagents that are not commonly available [1]. Diagnosis using RT-PCR and electrochemical measurement also takes a long time [10], and electrochemical measurement requires expensive equipment. By contrast, diagnosis by electrical measurement using capacitance is as short as 10 s and has high signal accuracy for the sample. Therefore, it is a suitable platform for use in Zika virus detection.

Several studies have focused on aptamers in biosensor fabrication as a useful bioprobe [11, 12]. When compared to antibodies, the use of aptamers for biosensor has several advantages. For example, aptamers can be manufactured by chemical synthesis that reduces manufacturing cost, and no animal testing is required. And the production speed of chemical synthesis of aptamer is relative faster than antibody production from animal experiment [13]. Moreover, specific functional groups can be easily introduced in aptamers [14] and several studies have reported aptasensors for virus detection [11, 12, 15]. Thus, the aptamer-based virus biosensor development is highly required to establish the zika virus diagnosis system for preventing the fast spreading of zika virus in the future. To solve this problem, the present study firstly synthesized the zika virus aptamer for biosensor application.

The Zika virus aptamer was synthesized using the SELEX technique and applied to a capacitance-based biosensor composed of DNA aptamer/MXene on Au micro-gap electrode (AuMGE) to detect Zika virus in human serum samples. The chemically produced aptamer is a single-stranded nucleic acid that can specifically bind to the Zika virus envelope proteins. MXene was used to increase the detection sensitivity. MXene is a plate-shaped nanomaterial composed of transition metal carbides and nitrides, and exhibits easy processability, high charge storage capacity, high mechanical strength, and excellent electron/ion conductivity [16]. In addition, MXene has proven to be a highly sensitive and selective sensing platform for sensing applications [17]. When the target protein binds to the aptamer to the fixed MXene of AuMGE under AC conditions, the capacitance changes, and the LCR meter can measure capacitance with high sensitivity. This method is more cost-effective than other electrical signal analysis equipment [16].

**Fig. 1:**
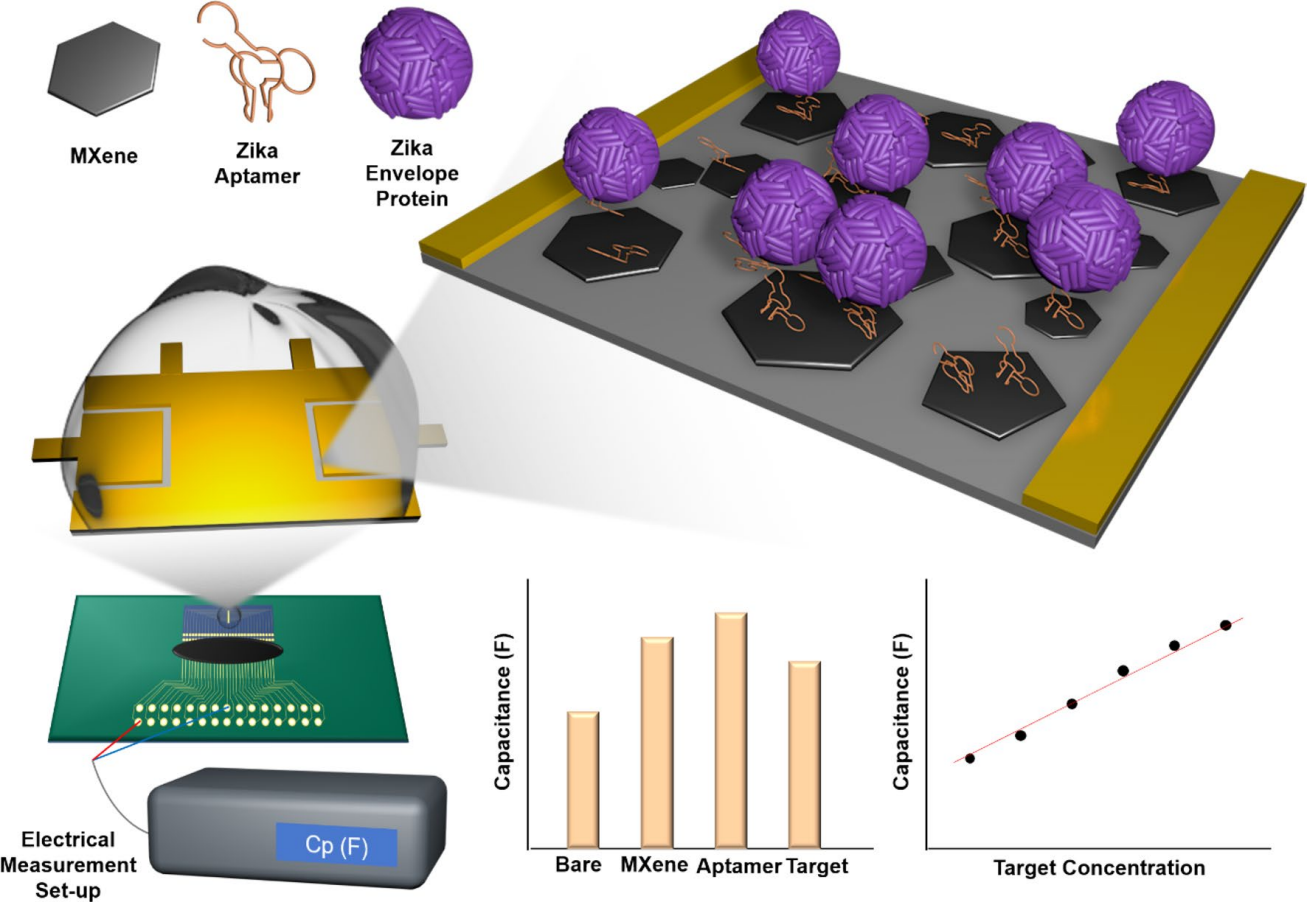
Schematic image of the fabricated biosensor using the aptamer/MXene for zika virus detection

Since the AuMGE provides a small active area, it is possible to measure with a small amount (10 μl) of sample, and the test can be repeated up to 28 times with one electrode. Furthermore, MXene-based biosensors are utilized as effective transducers for immobilizing biological receptors on surfaces and have proven to be highly sensitive and selective sensing platforms [18–20]. Figure 1 shows a schematic image of the proposed biosensor.

## Materials and methods

### Materials

For performing SELEX, the screening of Zika envelope aptamer was carried out by procurement of XELEX DNA Core kit from EURx (Poland) based on previous research [13]. The DNA library in the kit consists of the sequence 5′-TGA CAC CGT ACC TGC TCT-N40-AAG CAC GCC AGG GAC TAT-3′, the forward primer is 5′-TGA CAC CGT ACC TGC TCT-3′, and the reverse primer is sequence of 5′-ATA GTC CCT GGC GTG CTT-3′. PCR master mix containing DNA polymerase, Zika aptamer and thiol-modified Zika aptamer were purchased from Bioneer (South Korea). The thiol-modified Zika envelope aptamer consists of the sequence SH-5′-TGA CAC CGT ACC TGC TCT AGT GCG CAC TGA ACG ATC CTG CGT CAA GTT CAA GGT TGT GAA GCA CGC CAA GGG ACT AT-3′. The sequence information of aptamers obtained by performing SELEX is written in Table 1. Zika envelope protein was purchased from Sino biological (China), Sulfo-NHS-LC-Biotin was purchased from Thermo Fisher (USA), Aminopropyltriethoxysilane (APTES), 6-Mercaptohexanoic acid (6-MHA), and Mercaptopropyltriethoxysilane (MPTES) were purchased from Sigma-Aldrich (USA) and Streptavidin magnet beads were purchased from Genscript (USA). The AuMGE was manufactured by SNI technology (South Korea) based on previous research, and the printed circuit board (PCB) combined with the electrode was airlifted from Pulax Korea (South Korea).

**Table 1:** List of Zika envelope aptamers screened by SELEX process

Aptamer	Sequences	Prediction dG
Zika-07	TGACACCGTACCTGCTCTAGTGCGCACTGAACGATCCTGCGTCAAGTTCAAGGTTGTGAAGCACGCCAAGGGACTAT	− 8.78
Zika-09	ATAGTCCCTTGGCGTGCTTGATGCCCGGAAAATAAAAATCACAAGAACTACCCCAGCCCAGAGCAGGTACGGTGTCA	− 6.67
Zika-17	TGACACCGTACCTGCTCTCGTACAGCGAGCCGTTCTAGCTACTGGATTGAGGGTCACGAAGCACGCCAAGGGACTAT	− 6.66
Zika-25	TGACACCGTACCTGCTCTAAGCACGCCAAGGGACTATAGGTTGACACCGTACCTGCTCTAAGCACGCCAAGGGACTAT	− 7.11

### Zika envelope aptamer SELEX

The development of Zika envelope-specific aptamers is performed based on the SELEX procedure. In this study, the existing SELEX procedure, which is performed based on the binding of a His tag-existing target protein with a cobalt bead, was modified. In the sample preparation step, the reaction between the target protein and Sulfo-NHS-LC-biotin binds biotin to the protein surface based on the S–S bond. Proteins modified with biotin The sub-ssDNA library and DNA stabilization buffer used in the experiment were purchased and used by the XELEX DNA Core kit (EURx, Poland).

In this study, the SELEX process consists of four steps: protein sample preparation, binding of ssDNA to the target protein, capture and isolation of ssDNA, and amplification through PCR. The concentration of target protein, reaction temperature and time, and PCR conditions were set based on the optimization performed in previous studies [12]. Additional file 1: Fig. S1 shows a schematic diagram of the main steps of SELEX.

In the sample pretreatment step, 50 μl of Zika envelope protein diluted to a concentration of 0.25 mg/ml and 10 μl of 2 mM Sulfo-NHS-LC-Biotin (Thermo Fisher, USA) were stirred at room temperature at high speed for 1 h to biotinylate the Zika envelope surface. Unreacted biotin was removed by centrifugation for 20 min at 4000 rpm using a 3000 NMWL Amicon centrifuge filter (Merck, USA). Next, the protein on the top of the filter was recovered, 100 μl of Streptavidin mag net beads (Genscript, USA) were added, and the mixture was stirred for 1 h. Subsequently, the unreacted Streptavidin magnet beads were washed out 3 times using SELEX buffer containing NaCl, KCl, Tris, and MgCl_2_, which are components of the kit. The DNA library in the kit consists of the sequence 5′-TGA CAC CGT ACC TGC TCT-N40-AAG CAC GCC AGG GAC TAT-3′; the forward primer is 5′-TGA CAC CGT ACC TGC TCT-3′, and the reverse primer is 5′-ATA GTC CCT GGC GTG CTT-3′. The protein-bound streptavidin magnet beads and 200 μl of DNA library diluted with SELEX buffer were reacted for 1 h, and the reaction product was washed 5 times using the buffer. Then the beads bound with DNA were collected using a magnet. The Zika envelope protein was denatured by heat-treating the sample in an 80 °C thermostat for 10 min. Finally, beads and denatured proteins were removed by centrifugation, and template DNA from the supernatant was collected.

PCR master mix containing DNA polymerase, Zika aptamer, and thiol-modified Zika aptamer were purchased from Bioneer (South Korea). Amplification of template DNA through PCR was performed as follows: denaturation at 94 °C for 3 min; annealing at 55 °C for 30 s; and amplification at 72 °C for 1 min. The PCR cycle was repeated 10 times.

From the second SELEX round, it is performed using the ssDNA pool amplified by the PCR process in the previous round instead of the sub-ssDNA library. To confirm the reactivity of the amplified ssDNA pool and the target Zika envelope protein, 8% TBE-PAGE was performed in rounds 4 and 10 using the ssDNA pool. The binding of the ssDNA pool to the zika envelope protein in round 10 significantly changed the DNA band compared to the control (Additional file 1: Fig. S2). Then, the final ssDNA pool was purified using a 1% agarose gel, followed by sequencing through unidirectional RCA and 50 vector cloning at Solgent (South Korea).

Among the 50 sequences, duplicate sequences and sequences with unclear sequences due to sample contamination were excluded. And the most stable four nucleotide sequences with the smallest dG were finally selected, and the sequence information is presented in Table 1.

Secondary structures and dGs of the four candidate sequences selected in session 2.2 were predicted prior to measuring binding affinity. The secondary structure of the predicted sequence is shown in Additional file 1: Fig. S3, assuming a salt condition of 140 mM Na^+^ ion and 5 mM Mg^2+^ ion and a room temperature condition of 25 °C through mFold application (www.unafold.org). In addition, the dG values for the secondary structures are shown in Table 1. Based on the dG value of the sequence, the Zika-7 sequence and the Zika-25 sequence with the lowest dG values were selected and bound to the Zika envelope. Proteins were identified through 8% TBE PAGE.

### Validation of binding affinity of Zika envelope aptamer

Before measuring binding affinity, the secondary structure and dG of the four candidate sequences selected in session 2.2 were predicted. the secondary structure of the predicted sequence, assuming the salt conditions of 140 mM Na^+^ ions and 5 mM Mg^2+^ ions and the room temperature conditions of 25 °C through the mFold application (www.unafold.org), is shown in Additional file 1: Fig. S3. In addition, the dG values for the secondary structure are written in Table 1. Based on the dG value of the sequence, the Zika-7 sequence, and the Zika-25 sequence having the lowest dG value were selected and binding to the Zika envelope protein was confirmed through 8% TBE PAGE.

Binding affinity measurements are performed with a bead-based fluorescence binding assay. Fluorescein phosphoramidite (FAM), a fluorescent substance, was added to the 5' ends of the Zika-7 sequence and the Zika-25 sequence, and diluted to 3 nM, 30 nM, 300 nM, 1 μM, and 1.5 μM. Then, each concentration of aptamer was incubated with a magnet bead to which Zika envelope protein was fixed after the sample pretreatment step of Session 2.2 for 1 h. Afterwards, the magnet bead was washed 5 times with a washing buffer to remove unbound aptamers, denatured proteins by heat treatment at 80 °C, and centrifuged to recover aptamers. The collected fluorescent aptamers were measured for absorbance at 480 nm and emission at 528 nm using a Bio Tek Synergy LX multimode reader (USA). The fluorescence intensity was plotted according to the aptamer concentration, and Kd was calculated through a calibration curve based on the adsorption isotherm equation.

### Bioprobe immobilization

MXene, synthesized according to a previous protocol [18], was diluted to 1 mg/ml with deionized water (DIW). The AuMGE was manufactured by SNI technology (South Korea) based on previous research [19], and the AuMGE surface was ultrasonically cleaned with acetone for 10 min. Then, 10 μl of 5% aminopropyltriethoxysilane (Sigma-Aldrich, USA) was applied to AuMGE for 11 min to form an NH_2_ layer, and silane was activated by heating in an oven at 70 °C for 1 h. Thiol-modified (SH–) groups were generated by reacting 10 μl of 6-mercaptohexanoic acid (6-MHA, Sigma-Aldrich, USA) and activated AuMGE for 1 h. Then, 10 μl of 1 mg/ml MXene was added and maintained at room temperature for 12 h for the reaction to occur. Finally, 10 μl of 5% mercaptopropyltriethoxysilane (MPTES, Sigma-Aldrich, USA) was applied for 11 min to create a SH group-modified layer.

SH group-modified Zika virus envelope protein aptamer was diluted to 1 μM with DIW, and 10 μl was reacted with the SH layer of AuMGE for 3 h to deposit the aptamer and bind it through self-assembly. All reactions were carried out at room temperature, and the residue was removed with DIW and N_2_ gas.

### Surface investigation by atomic force microscopy

Characterization of biomolecules immobilized on Au electrode surfaces was investigated for SiO_2_ bare electrodes, SiO_2_/MXene electrodes, and SiO_2_/MXene/Aptamer/Zika envelope immobilized electrodes in non-contact mode using atomic force microscopy (AFM; XE7, Park systems, Korea). The cantilever used for the investigation was PPP-NCHR (Park systems, Korea) and a silicon tip with a resonant frequency of 330 kHz and a spring constant of 42 N/m was selected. AFM measurement conditions were established based on a previous study [20].

### Capacitance detection

Capacitance was measured using an LCR meter (E4980AL, Keysight technologies, USA) controlled by LabView program. The probe of the LCR meter was connected to a socket on the PCB (Pulax Korea, South Korea) where AuMGE was mounted. Capacitance was measured at 10 mV for 10 s at 1 MHz with reference to the previous study [19].

## Results and discussion

### Investigation of reactivity of the Zika envelope aptamer

**Fig. 2:**
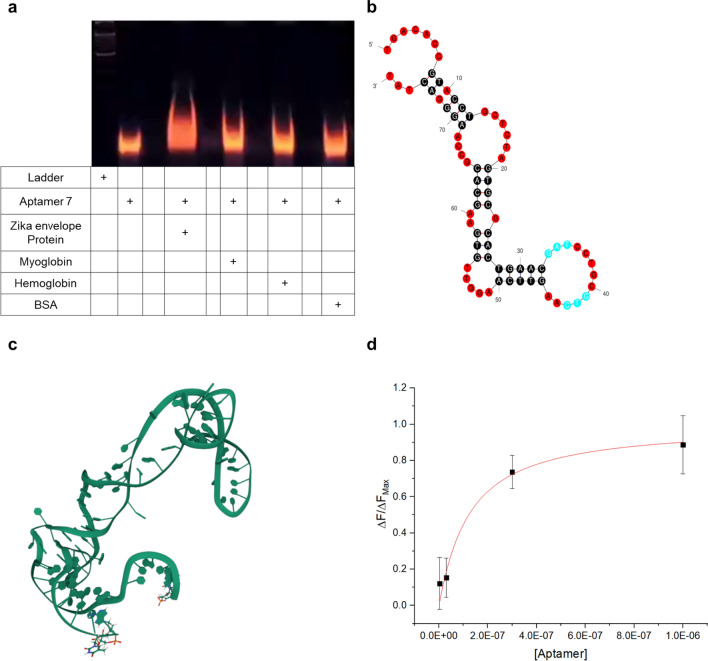
**a** 8% TBE-PAGE result of Zika Aptamer; **b** Expected 2D structure of Zika aptamer base; **c** Expected 3D structure of Zika aptamer base; **d** Binding affinity of Zika aptamer

After sequencing, 50 aptamers were obtained by SELEX. The secondary structure of the Zika envelope aptamer was predicted by mFold at 25 °C with a buffer composition of 140 mM Na^+^ ions and 5 mM Mg^2+^ ions. Based on the predicted dG values, two aptamers were selected. and binding was determined using 8% native-TBE PAGE binding test. Figure 2a–c and Additional file 1: Fig. S4a, b show the predicted structures of aptamers (Zika-7 and Zika-25) and PAGE results. The 3D model of the aptamer in Fig. 2c was predicted with a 3dRNA/DNA web server based on the dot-bracket format of the aptamer secondary structure. For the two aptamer sequences, lane 2 contained only the aptamer, and lane 4 contained the aptamer and the target material, Zika envelope protein, which showed band changes according to the binding of the aptamer; myoglobin was used as the control. Lane 6 showed only a slight difference compared to lane 2. When the hemoglobin was added to zika aptamer, there in no change in the gel (lane 8), also, the fabricated zika virus aptamer didn’t bind with albumin (lane 10). Thus, the synthesized aptamer can bind to the envelope protein of the zika virus specifically.

Moreover, a bead-based fluorescence binding assay was performed to confirm the dissociation constant of the aptamer. After FAM was introduced at the 5′ end of the Zika-7 sequence and the Zika-25 sequence, samples were incubated at different concentrations. The fluorescence intensity according to the collected aptamer concentration is graphically shown in Fig. 2d and Additional file 1: Fig. S4c. The K_d_ of the aptamer was calculated based on the fluorescence intensity and the protein concentration according to the aptamer concentration using the following isothermal adsorption Eq. (1) [21].

1 $$\frac{\Delta{\text{F}}}{\Delta{{\text{F}}_{\text{Max}}}}\text{=}\frac{{\text{C}}_{\text{Zika E}}}{{\text{K}}_{\text{d}}{+}{\text{C}}_{\text{Zika E}}}.$$

The Kd of Zika-7 aptamer is 106.9 ± 1.9 nM and that of Zika-25 aptamer is 226.1 ± 9.6 nM. Low Kd values are suitable for biosensor construction as bioprobes. Based on the results, the sensor was constructed using Zika-7 aptamer, and a thiol functional group was added to the 5′ end of Zika-7 aptamer before use.

### Surface investigation of Zika envelope/aptamer on MXene

**Fig. 3:**
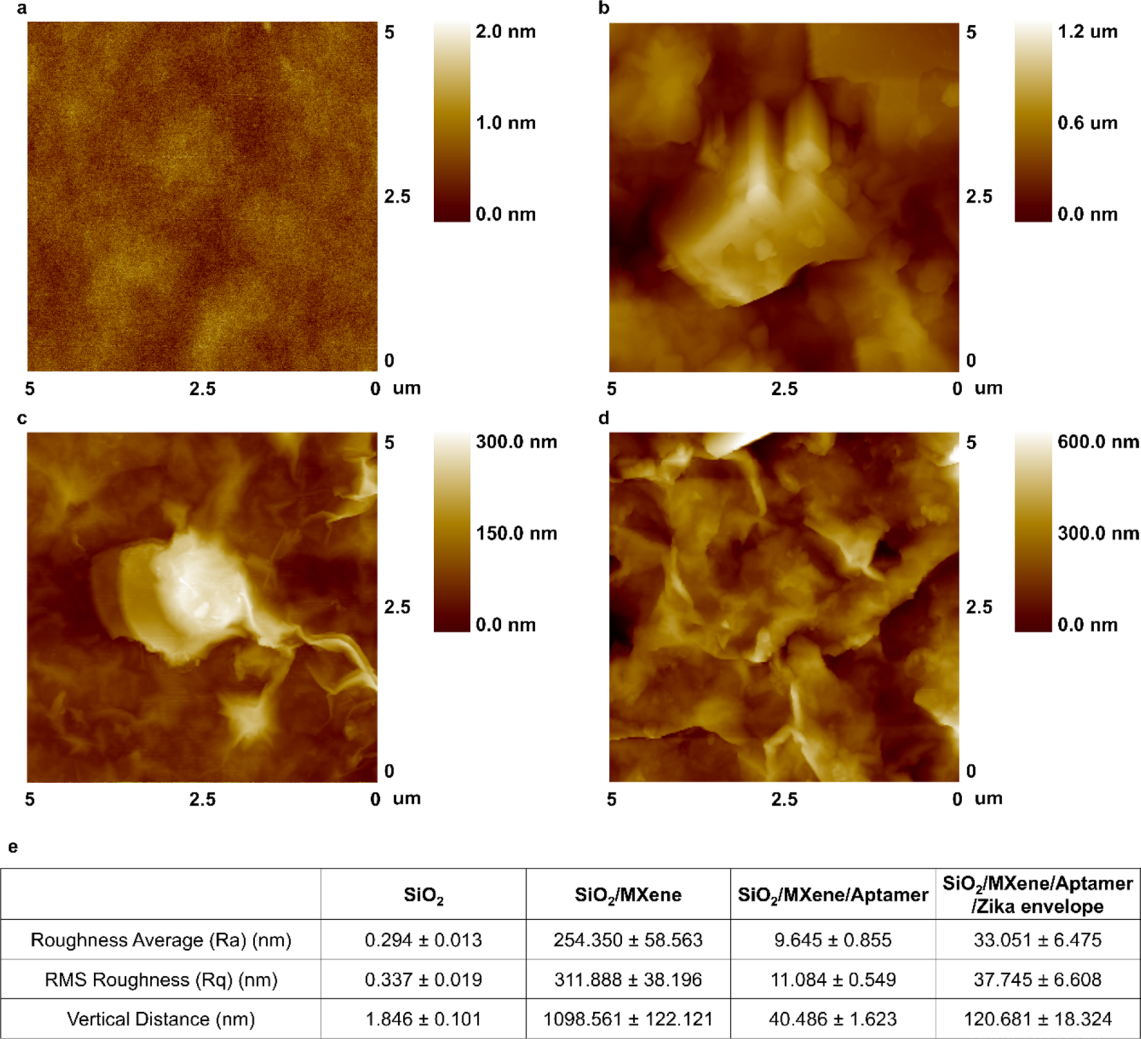
**a** AFM image of SiO_2_ substrate; **b** AFM image of MXene on SiO_2_ substrate; **c** AFM image of MXene, Aptamer on SiO_2_ substrate; **d** AFM image of MXene, Aptamer, and Zika virus envelope protein on SiO_2_ substrate; **e** Surface Analysis of SiO_2_, MXene, Aptamer, Zika virus envelope protein using Roughness average, RMS and vertical distance

For investigating the surface morphology of biofilm, AFM experiment was carried out. In addition to, the AFM-based surface rougness investigation provided the useful information for determining the immobilization. We analyzed the roughness average (Ra), root-mean-squared (RMS) roughness (Rq), and vertical distance (Vd) of the each sample (Fig. 3a–e). The surface of the SiO_2_ bare substrate is shown in Fig. 3a, and no other materials were observed on the surface of the substrate. The Ra value of the SiO_2_ substrate was 0.294 ± 0.013 nm, and the Rq was 0.337 ± 0.019 nm. Figure 3b shows the surface of the MXene-modified substrate on the SiO_2_ substrate. The Ra of the MXene-immobilized substrate was 254.350 ± 58.563 nm, Rq was 311.888 ± 38.196 nm, and Vd was 1098.561 ± 122.121 nm. From the AFM image and Vd value, it can be confirmed that the cube-shaped MXene is fixed and distributed on the surface. Figure 3c shows an image of an aptamer bound to a surface on which MXene is immobilized. Due to the aggregation, it was possible to confirm the form of agglomerated aptamers. In the image, in the region where the aptamer was fixed, Ra was 9.645 ± 0.855 nm, Rq was 11.084 ± 0.549 nm, and Vd was 40.486 ± 1.623 nm, indicating a large difference compared to MXene. Figure 3d shows the AFM measurement results of the surface of the aptamer-immobilized MXene substrate continuously reacted with the envelope protein. Similar to Fig. 3c, the protein is immobilized on the surface in the form of a lump. The substrate has Ra of 33.051 ± 6.475 nm, Rq of 37.745 ± 6.608 nm, and Vd of 120.681 ± 18.324 nm. The successful immobilization of self-assembled aptamers to MXene and proteins was confirmed by the differences in Ra, Rq, and Vd values at each step. Additional file 1: Fig. S5a–c show AFM images over a larger surface area.

### Investigation of electrical characteristics of the AuMGE electric sensor

The AuMGE fabricated in this study consists of a large-area counter electrode and 28 electrodes with a gap of 5 μm. Each electrode is bound to a socket connected to the PCB, and for this purpose, the length of the wire connecting the electrode and the pad has a certain difference. As shown in Additional file 1: Fig. S6, as the measuring electrode moves away from the counter electrode, the socket number increases and the capacitance of the bare electrode decreases due to parasitic capacitance [19]. The capacitances of the left and right symmetric electrodes have the same value. Through this, it is easy to detect disconnection of electrodes or defects, and it has the advantage of easy repeat measurement as multiple electrodes are concentrated in one biosensor.

The manufactured bare electrode is immobilized on the electrode surface in the order of MXene, Aptamer, and Protein, and the measured capacitance changes depending on the material to be fixed. The change in capacitance is defined using Eq. (2) consisting of the dielectric constant of the medium, dielectric constant of the free space, area of the electrode, and distance between the electrodes based on previous research:

2 $$ C = \varepsilon \varepsilon_{0} A/d, $$

**Fig. 4:**
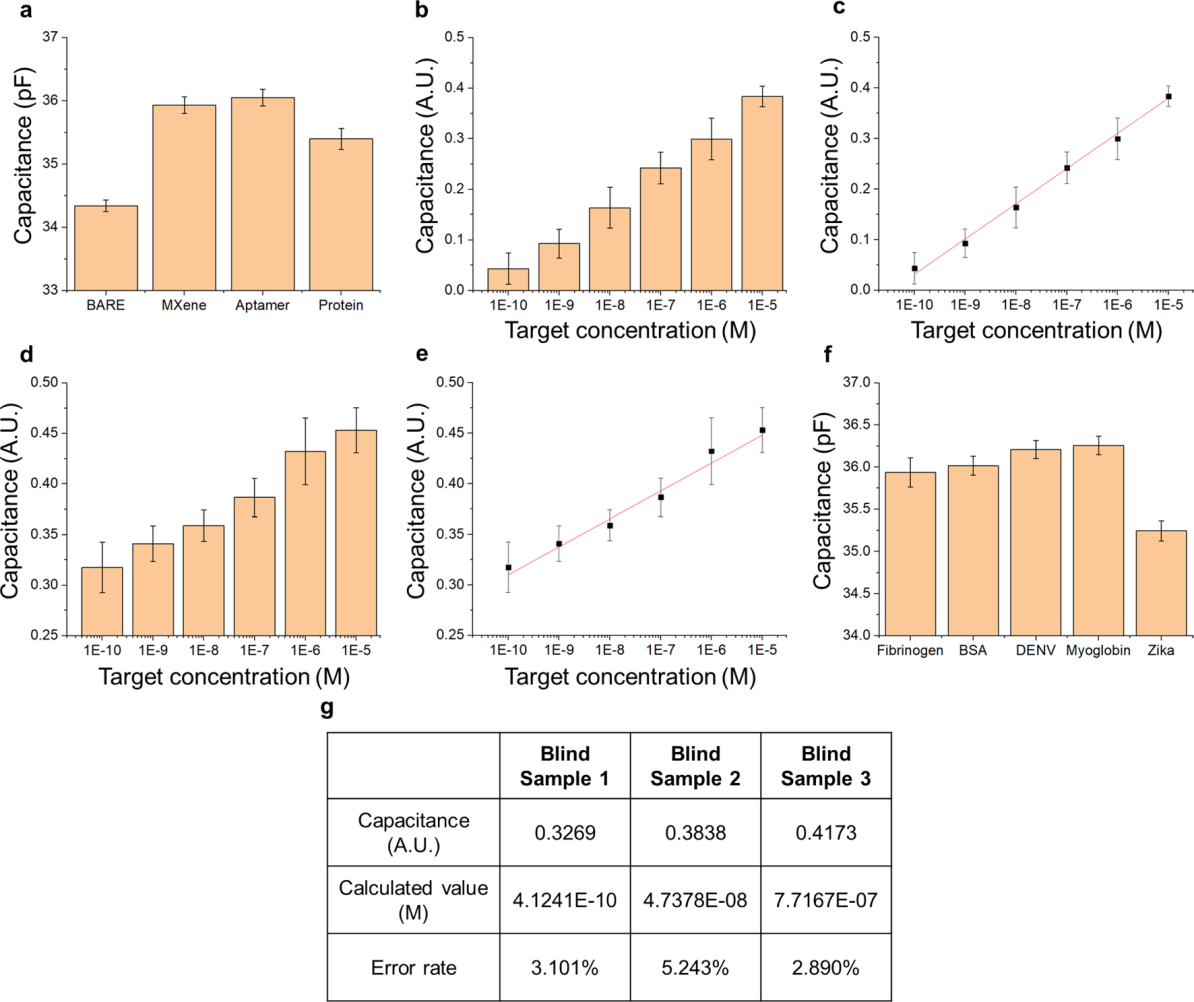
**a** Capacitance trend according to the binding step; **b** Capacitance trend by zika virus envelope protein concentration in DIW; **c** Calibration curve according to zika virus envelope protein concentration in DIW; **d** Capacitance trend by zika virus envelope protein concentration in 10% human serum; **e** Calibration curve according to zika virus envelope protein concentration in 10% human serum; **f** Selectivity of the fabricated biosensor; **g** Biosensor blind test result

*ε* is the dielectric constant between the electrodes, *ε*_*0*_ is the permittivity of free space, *A* is the area of the electrodes, and *d* is the distance between the electrodes. Figure 4a shows the change in capacitance at each stage. The capacitance increases immediately after MXene and aptamer fixation and tends to decrease immediately after aptamer and protein binding. This is because the protein is immobilized on the electrode surface, blocking the electrode surface and water molecules having a relatively high dielectric constant, thereby reducing the capacitance value.

### Sensor performance evaluation and Zika envelope protein serum test

Sensor performance evaluation was performed by diluting the Zika envelope protein concentration from 100 pM to 10 μM in six steps. As shown in Fig. 4b, the signal from the sensor tends to decrease as the concentration of the sample decreases. This is because the dielectric properties of the electrode changes according to the concentration of Zika envelope protein immobilized on the electrode surface; therefore, a high concentration of Zika envelope results in a greater change in dielectric properties. The detection limit of the sensor was calculated by creating a trend line based on the signal for each concentration. As shown in Fig. 4c, the sensor’s slope is 0.069 ± 0.001, and the sensor’s LOD calculated based on the graph with intercept 0.728 ± 0.014 is 93.14 pM (At S/N = 3).

The LOD was calculated using the Zika envelope protein sample diluted with 10% human serum. In the same manner as the experimental conditions performed previously, measurements were carried out in six steps, 10 times each, in the concentration range from 100 pM to 10 μM, and the measured results are shown in Fig. 4d–e. Under the condition of 10% human serum, the sensor slope was 0.027 ± 0.002, the intercept was 0.585 ± 0.016, and the sensor LOD was 38.14 pM. In addition, to confirming the selectivity of the sensor, detection was measured in the presence of major interfering substances in the blood such as hemoglobin, myoglobin, fibrinogen, and bovine serum albumin and Dengue virus envelope proteins. The experimental results are shown in Fig. 4f. Our sensor showed a high signal in response to the Zika envelope but showed no significant reactivity with other proteins. Finally, a blind test was performed using Zika envelope protein diluted in 10% serum. Figure 4g shows the concentration derived from the calibration curve for a sample of Zika at a random concentration. Our sensor can detect Zika envelope proteins with an error rate of within 3.101%, 5.243%, and 2.890%. Recently, research with excellent linear detection range and LOD for Zika virus detection has been conducted. Table 2 presents the results of these studies. However, our sensor was the first to select a Zika envelope-specific aptamer and introduce it as a probe, and the aptamer has the advantage of reducing the time and cost required for production compared to the antibody. In addition, by introducing an electrical measurement method, rapid detection within 10 s is possible under low voltage conditions with little damage to biomolecules. Therefore, our aptamer and detection platform may be utilized in the field of Zika research in the future.

**Table 2:** Biosensor comparison table for Zika virus detection

Probe	Detection method	Target material	Detection range	LOD	Refs.
Antibody	EC	E protein	10 pM–1 nM	10 pM	[8]
Antibody	E	NS1 protein	0.1–100 ng/ml	0.1 ng/ml	[21]
Antibody	E	NS1 protein	N/A	0.45 nM	[22]
Antibody	EC	NS1 protein	0.1–100 ng/ml	1 pg/ml	[23]
Surface Imprinted Polymers	EC	prM-E protein	1 × 10^–3^–1 × 10^2^ PFU/ml	2 × 10^–3^ PFU/ml	[24]
ssDNA	EC	DNA	1 pM–10 mM	0.82 pM	[25]
Peptide aptamer	FICT	E protein	0.15–10.92 ng/ml	0.15 ng/ml	[26]
Aptamer	E	E protein	100 pM–10 μM	38.14 pM	This work

## Conclusion

This study presented the first Zika virus aptamer that successfully binds the envelope protein of the Zika virus. Moreover, the prepared aptamer was introduced to the electrical biosensor as the bioprobe for Zika virus detection with MXene and AuMGE. In this study, the Zika virus aptamers with high reactivity with Zika envelope protein were selected through a screening process known as SELEX. MXene is a nanoparticle with a high electrical conductivity that is fixed to the electrode gap to expand the reaction area of the electrode and improve sensitivity. The proposed biosensor, based on a combination of the aptamer, MXene, and AuMGE can detect a Zika virus envelope protein at a concentration of 38.14 pM within 10 s in 10% human serum conditions. Moreover, the blind test results showed the prepared aptasensor can detect Zika virus well in undefined samples. Compared with previous studies (Table 2), the authors developed a high-sensitivity electrical aptasensor with a wide detection range and low detection limit. In addition, Zika virus aptamer was used as an alternative to the existing antibody, which can be used as a more powerful tool in various fields along with the detection system.

## Supplementary Information

**Additional file 1: Fig. S1** Schematic diagram of SELEX Process. **Fig. S2** 8% TBE PAGE result of 10 round ssDNA pool. **Fig. S3** Structures of candidate aptamers prepared in Table. 1 **a** Zika -07 aptamer; **b** Zika-09 aptamer; **c** Zika-17 aptamer; **d** Zika-25 aptamer. **Fig. S4** **a** 8% TBE PAGE result of Zika 25 Aptamer; **b** 2D structure of Zika 25 aptamer base; **c** Binding affinity of Zika aptamer.

## Data Availability

The authors have no data to share since all data are shown in the submitted manuscript.
