# Correction to: Selection of DNA aptamer and its application as an electrical biosensor for Zika virus detection in human serum

**DOI:** 10.1186/s40580-022-00340-8

**Published:** 2022-10-31

**Authors:** Goeun Park, Myoungro Lee, Jiatong Kang, Chulwhan Park, Junhong Min, Taek Lee

**Affiliations:** 1 Department of Chemical Engineering, Kwangwoon University, 20 Kwangwoon-Ro, Nowon-Gu, Seoul, 01897 Republic of Korea; 2 School of Integrative Engineering, Chung-Ang University, Heukseok-Dong, Dongjak-Gu, Seoul, 06974 Republic of Korea

## Correction to: Nano Convergence (2022) 9:41 http://doi.org/10.1186/s40580-022-00332-81

Following publication of the original article [1], the authors noticed that the repetition texts occurred in Acknowledgement and Funding sections. Hence, the following funding section has been removed from the original publication by publishing this erratum.

Acknowledgements

This work was supported by the National Research Foundation of Korea(NRF) grant funded by the Korea government (MSIT) (No. 2021R1C1C1005583) and by Korea Environment Industry & Technology Institute (KEITI) through the program for the management of aquatic ecosystem health, funded by Korea Ministry of Environment (MOE) (2020003030001) and by the Chung-Ang University Young Scientist Scholarship in 2020.

The original article [1] has been updated.
