# Supplementary material for: Selection of DNA aptamer and its application as an electrical biosensor for Zika virus detection in human serum
Source: Nano Converg. 2022 Sep 10;9:41. doi: 10.1186/s40580-022-00332-8 (PMC9463662; doi:10.1186/s40580-022-00332-8)
Supplement: Supplementary file 1 — Additional file 1: Fig. S1 Schematic diagram of SELEX Process. Fig. S2 8% TBE PAGE result of 10 round ssDNA pool. Fig. S3 Structures of candidate aptamers prepared in Table. 1 a Zika -07 aptamer; b Zika-09 aptamer; c Zika-17 aptamer; d Zika-25 aptamer. Fig. S4 a 8% TBE PAGE result of Zika 25 Aptamer; b 2D structure of Zika 25 aptamer base; c Binding affinity of Zika aptamer. [file 40580_2022_332_MOESM1_ESM.docx]

Supporting Information

Selection of DNA aptamer and its application as an electrical biosensor for Zika virus detection in human serum

Goeun Park^1,†^, Myoungro Lee^1,†^, Jiatong Kang^2^, Chulwhan Park^1^, Junhong Min^2,*^, Taek Lee^1,*^


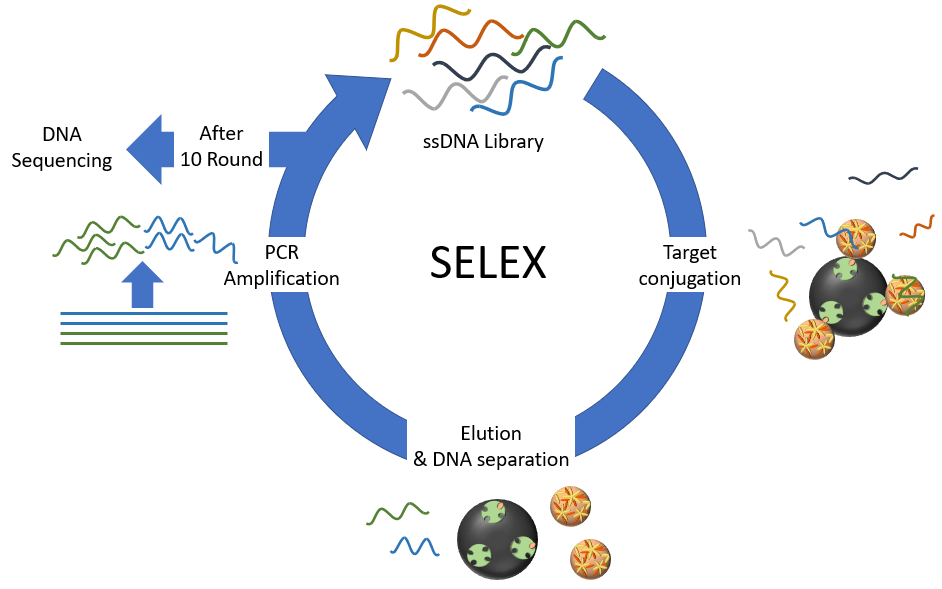


Fig. S1 Schematic diagram of SELEX Process


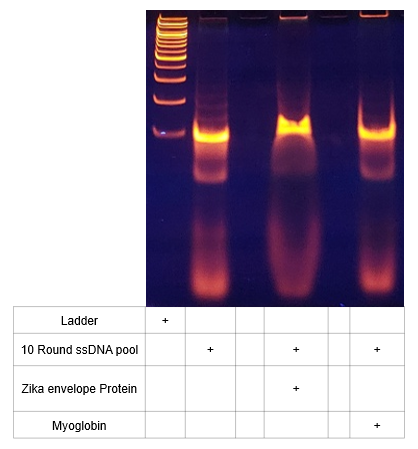


Fig. S2 8% TBE PAGE result of 10 round ssDNA pool


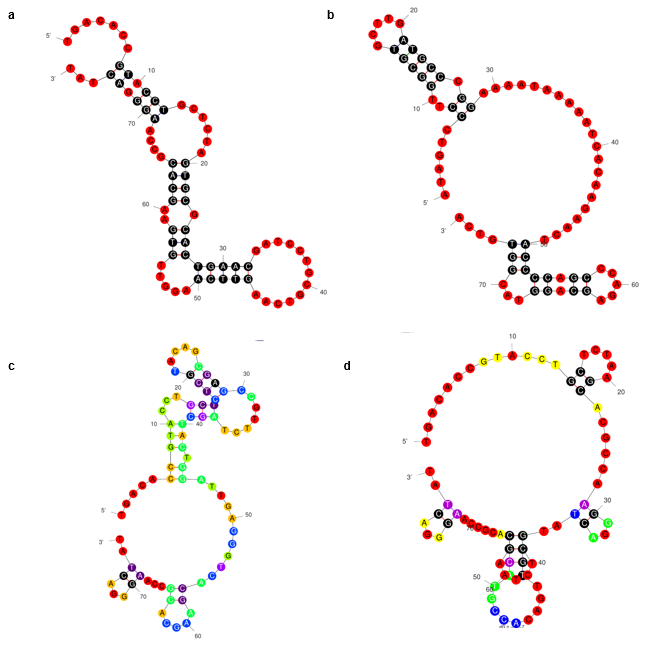

Fig. S3 Structures of candidate aptamers prepared in Table. 1 **a** Zika -07 aptamer; **b** Zika-09 aptamer; **c** Zika-17 aptamer; **d** Zika-25 aptamer


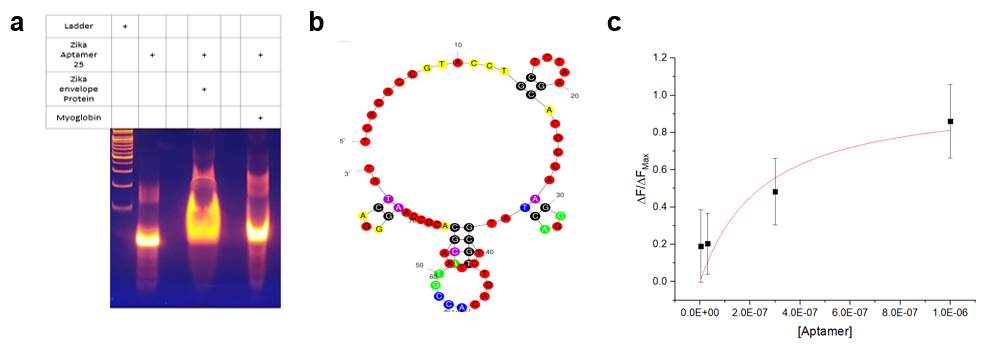


Fig. S4 **a** 8% TBE PAGE result of Zika 25 Aptamer; **b** 2D structure of Zika 25 aptamer base; **c** Binding affinity of Zika aptamer


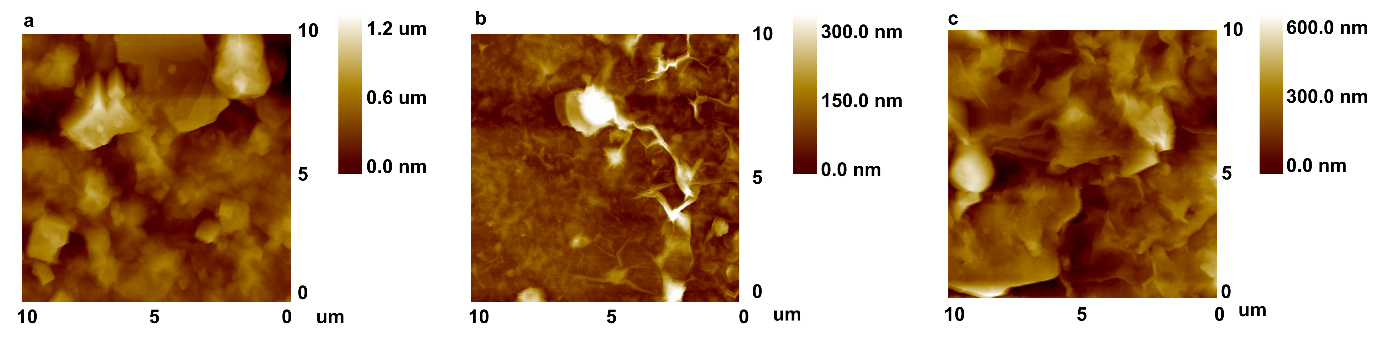
Fig. S5 AFM image measured with a larger area **a** MXene on SiO_2_ substrate; **b** MXene, Aptamer on SiO_2_ substrate; **c** MXene, Aptamer, and Zika virus envelope protein on SiO_2_ substrate

 Fig. S6 Capacitance trend by AuMGE socket
